# Treatment of ureteral stones: A prospective randomized controlled trial on comparison of Ho:YAG laser and pneumatic lithotripsy

**DOI:** 10.4103/0970-1591.39549

**Published:** 2008

**Authors:** Robab Maghsoudi, Mohsen Amjadi, Davood Norizadeh, Hassan Hassanzadeh

**Affiliations:** Department of Urology, Imam Hospital, Tabriz University of Medical Sciences, Tabriz, Iran

**Keywords:** Ho: YAG laser, pneumatic lithotripsy, ureteral stones

## Abstract

**Objectives:**

To study the treatment of ureteric stones by HO:YAG laser lithotripsy and pneumatic lithotripsy and to evaluate the results of the two treatment modalities to assess effectiveness and complications.

**Materials and Methods:**

Over 1-year period, a total of 79 patients with 82 ureteral stones were randomized into two groups. In group 1 (39 cases with 41 ureteral stones) ureteroscopic HO:YAG laser lithotripsy was performed using a rigid 8 Fr-ureteroscope (LL group). In group 2 (40 cases with 41 ureteral stones) pneumatic lithotripsy was performed in like manner. Efficacy safety and complications in both groups were analyzed.

**Results:**

A total of 79 patients with 82 calculi were treated. Two cases in LL group and one in PL group had bilateral ureteral stones. Mean stone size was 12.07 mm in LL group and 10.2 mm in PL group. Stones located in lower ureter in 30 cases on LL group and 29 cases in PL group. Proximal migration of stone occurred in 1 case on LL group and in 3 cases on PL group. Successful fragmentation occurred in 37 cases on LL group and in 30 cases on PL group. Stone-free rate after 1 month in the base of Kidney Ureter Bladder (KUB) and sonography was 95% in LL group and 80.5% in PL group. Ureteral perforation, urinoma, and urosepsis were not seen in both groups.

**Conclusion:**

HO:YAG laser has advantages over PL in high efficacy of stone fragmentation and a low-retrograde migration of ureteral stone treatment. Other complication of ureteral stone treatment with LL and PL are the same and very rare.

## INTRODUCTION

Endoscopic management of ureteral calculi is one of the most important therapies in academic centers.[1] Different kinds of lithotripters are used through the ureteroscope that revolutionized the treatment of ureteral calculi.[2] Two most common lithotripters that used in urologic fields are pneumatic and Ho:YAG laser. Pneumatic lithotripsy is more popular among the urologists because of its low cost, easy setup, and high success rate.[3] Nevertheless, proximal migration of calculi may be a limiting factor of this method.[4] Ho:YAG laser is a reliable method for the treatment of urethral stones especially in proximal and impacted ureteral stones, but it is expensive and not available in most of the urologic centers.[5]

There are a few studies to comparison of these two techniques and the aim of this study is evaluation of the results of Trans Ureter Litotripsy (TUL) with these two methods according to their fragmentation, complication, proximal migration, and stone-free rates.

## MATERIALS AND METHODS

In a clinical trial study science February 2003 to February 2004, a total of 79 patients with 82 ureteral stones were randomized in two groups. In group 1 (39 cases with 41 ureteral stones), Ho:YAG laser lithotripsy was performed by a rigid 8.5-Fr Wolf ureteroscope (LL group) and in group 2 (40 cases with 41 ureteral stones), the procedure was performed with a pneumatic lithoclast (PL group) at the same size of ureteroscope.

Patients were included in the study when they had ureteral stone which not passed in 2 weeks, presence of hydronephrosis, and failed Extracorporeal Shock Wave Litotripsy (ESWL). Stones more than 1.5 cm were excluded. In all patients, urinalysis, urine culture, KUB, ultrasonography, and intravenous pyelography were performed before TUL. Preoperatively, all patients were received 1 g ampicillin and 80 mg gentamycin intravenously. The patients were placed in lithotomy position and cystoscopy was performed before the TUL. A guide wire was inserted into the ureteral orifice and ureteroscope (8.5-9 Fr wolf) was used in all patients. The stones were fragmented by a 0.8-mm Swiss lithoclast probe with the ballistic energy at a rate of 12 Hz or Ho:YAG laser (DECA 20 watt's Smart 2001) with end-firing probe 365 μm, 0.5-1 joule energy in frequency of 5-10 Hz. The indications for stenting were ureteral edema secondary to an impacted calculus, iatrogenic trauma, and residual stone burden. KUB and ultrasonography were done 1, 3, and 30 days after the procedure. Results were analyzed with Pearson χ^2^ and t-test.

## RESULTS

A total of 79 patients with 82 ureteral calculi were treated. Forty cases treated with pneumatic lithotripsy (PL group) and 39 cases with HO:YAG laser lithotripsy (LL group).

From 40 patients on PL group, 29 cases were males with mean age of 38.5 ± 6.4 years; and 11 cases were females with mean age of 42.5 ± 4.3 years, from 39 patients on LL group, 28 cases were males with mean age of 35.71 ± 41 years and 11 cases were females with mean age of 51.09 ± 5.1 years. Two cases in LL group and one in PL group had bilateral ureteral stones.

Mean stone size was 12.07 ± 2.1 mm in LL group and 10.2 ± 2.8 mm in PL group with a range of 6-15 mm in both groups. Stones located in lower ureter (below the iliac crest on the base of KUB) in 30 cases of LL group and 29 cases of PL group. In other patients stones location was in the upper ureter (above the iliac crest).

Proximal stone migration occurred in 1 case of LL group (2.4%) and in 3 cases of PL group (7.3%), that was insignificant (*P* = 0.6). Successful stone fragmentation occurred in 37 cases (90.2%) on LL group and in 30 cases (73.20%) on PL group. In Pearson χ^2^-test, this difference was significant (*P* = 0.46).

Stone-free rate after 1 month in the base of KUB and ultrasonography was 95% in LL group and 80.5% in PL group (*P* = 0.043) and this was significant also.

Mucosal tearing, ureteral perforation, urinoma, and urosepsis were not seen in both groups.

## DISCUSSION

Ureteral calculi that fail with conservative measures require intervention. Endoscopic lithotripsy is going on to be more evolve by progressing on technological advances in all direction and increase the efficacy and safety of ureteral stone treatment. Improvement and refinements in endoscopic lithotripters are expanded the patient population that need to treatment and included many patients with urinary calculi in this way.[1]

Minimally invasive technique for the treatment of ureteral stones should be evaluated from the standard points of efficacy and the ultimate success rate of the various procedures. These include the feasibility of the procedure, number of the sessions required to be the patient stone-free, complication rate, and the requirements to achieve the stone-free status. However, the popularity of the any particular method will be equally determined by the cost of stone removal, especially in developing countries.[6]

A variety of lithotripters can be used through an ureteroscope. Pneumatic and HO:YAG laser lithotripsy commonly used in majority of urologic centers.

This study compares the result of ureteral stone treatment with Ho:YAG laser and pneumatic lithotripsy as a randomized clinical trial.

The patient populations in the two groups were comparable in terms of the ureteral stones. Sex distribution in two groups was comparable, that is 29 males and 11 females in PL group and 28 males and 11 females in LL group.

Mean age of patients were 40.5 and 43.4 in PL and LL groups, respectively. This is general agreement with other reports in the literature.[6,7]

Stone size in both groups was 6-15 mm and mean stone size was 10.2 mm on PL group and 12.7 mm in LL group.

In Yinghao study, mean stone size was 11 ± 2.5 mm in PL group and 12 ± 2.3 mm in LL group.[3] In the other studies, mean size of stone generally was 9-16 mm.[1,7] This indicates that when we exclude stone larger than 15 mm in size for lithotripsy, in the majority of cases mean size of stone will be 10 ± 2 mm.

Under direct vision stone fragmentation with LL was better than PL, especially when stone surface was smooth and hard. In our study, stone fragmentation was 90.2% in LL group and 73.2% in PL group (*P* < 0.05). In Yinghao study, this was 95.7 and 69.7% for LL and PL groups, respectively.[3] However, Naqvi *et al*, reported stone fragmentation with PL was better than LL. Their laser was alexandrite laser.[6] Grasso reported 97% fragmentation rate with Ho:YAG laser.[1] Therefore, LL was better fragmentation rate especially in the setting for soft and hard stones, and fragments produced following LL were smaller than those produced by PL.

In this study, eight cases in PL and two in LL groups had fragments larger than 5 mm. After 1 month follow-up, three cases in PL and both of two cases in LL group, become stone free. However, retreatment performed in the rest of patients. Proximal stone migration is the most disadvantage of the pneumatic lithotripsy and reported in the 2-17% of cases.[7] In this study, proximal stone migration was 7.3% (three cases) in PL group and 2.4% (one case) in LL group. But this difference was not significant in the Fischer test (*P* = 0.6).

Proximal migration is related with dilatation of proximal ureter, size and hardness of stone, severity of stone impaction, and pressure of irrigation fluid.

Overall stone-free rate after 1 month without any retreatment procedure were 95.1% in LL and 80.5% in PL groups. This result is comparable with other studies.[1,3,5,6]

In our setting major complications such as ureteral perforation urinoma and urosepsis were not seen on both groups.

## CONCLUSION

It seems that Ho:YAG laser has advantages over pneumatic lithotripsy in high efficacy of stone fragmentation and a slightly low-retrograde migration in the treatment of ureteral stones.

Overall, stone-free rate in Ho:YAG laser lithotripsy is better than pneumatic lithotripsy. Other complications of ureteral stone treatment with Ho:YAG and pneumatic lithotripsy are the same and very rare. Further study is recommended in this field.
